# Urinary metabolomics study on the protective role of *Orthosiphon stamineus* in Streptozotocin induced diabetes mellitus in rats via ^1^H NMR spectroscopy

**DOI:** 10.1186/s12906-017-1777-1

**Published:** 2017-05-25

**Authors:** Amalina Ahmad Azam, Raghunath Pariyani, Intan Safinar Ismail, Amin Ismail, Alfi Khatib, Faridah Abas, Khozirah Shaari

**Affiliations:** 1 Laboratory of Natural Products, Institute of Bioscience, Universiti Putra Malaysia, 43400 Serdang, Selangor Malaysia; 2 Department of Biomedical Sciences, Faculty of Medicine and Health Sciences, Universiti Putra Malaysia, 43400 Serdang, Selangor Malaysia; 3 Faculty of Pharmacy, International Islamic University Malaysia, 25200 Kuantan, Pahang Malaysia

**Keywords:** *Orthosiphon Stamineus*, Java tea, Metabolomics, Diabetes mellitus, ^1^H NMR spectroscopy

## Abstract

**Background:**

*Orthosiphon stamineus* (OS) is a herb known in ethnomedicine for treating diabetes mellitus (DM). In this study, a ^1^H NMR based urine metabolomics tool has been used for the first time to identify the metabolic protective mechanism of OS in DM using Streptozotocin (STZ) induced experimental model in rats.

**Methods:**

Four different solvent extracts of OS, namely aqueous, ethanolic, 50% aqueous ethanolic and methanolic, at a dose of 500 mg/kg body weight (bw) were orally administered for 14 days to diabetic rats induced via intraperitoneal injection of 60 mg/kg bw STZ. NMR metabolomics approach using pattern recognition combined with multivariate statistical analysis was applied in the rat urine to study the resulted metabolic perturbations.

**Results:**

OS aqueous extract (OSAE) caused a reversal of DM comparable to that of 10 mg/kg bw glibenclamide. A total of 15 urinary metabolites, which levels changed significantly upon treatment were identified as the biomarkers of OSAE in diabetes. A systematic metabolic pathways analysis identified that OSAE contributed to the antidiabetic activity mainly through regulating the tricarboxylic acid cycle, glycolysis/gluconeogenesis, lipid and amino acid metabolism.

**Conclusions:**

The results of this study validated the ethnopharmacological use of OS in diabetes and unveiled the biochemical and metabolic mechanisms involved.

**Electronic supplementary material:**

The online version of this article (doi:10.1186/s12906-017-1777-1) contains supplementary material, which is available to authorized users.

## Background

Diabetes mellitus (DM) is a global menace, which ranks fourth among the diet-related non-communicable chronic diseases, after cardiovascular diseases, cancers and chronic respiratory diseases [1]. In 2014, an estimated 387 million people were identified to be diabetic worldwide, and the global health expenditure accounted for 612 billion US dollars [2]. Globally, around 80% of the DM population is represented by low and middle-income countries [2, 3], which could probably due to the unaffordable medical expenditures. One strategic approach to curb diabetes is, therefore, to promote the intake of regionally available herbs and functional food derivatives in the daily diet, either as food or beverages, which could afford a considerable amount of protection against this lifestyle disease. The herbs which could be used as such either in whole or in parts deserve special attention due to their feasibility for daily use, easy availability and relatively low cost.

Various cross-sectional surveys across different countries and regions of the world revealed that 17–72.8% patients rely on complementary and alternative medicine (CAM) methods in managing DM, either as a supportive measure along with the modern medicine or occasionally as a standalone therapy [4]. A cross-sectional study conducted in Malaysian population showed that the prevalence of CAM use in the management of DM was 62.5%, wherein among the use of herbs, *Orthosiphon stamineus* (OS) ranked second at the preference of 38.7% of the patients, to bitter gourd (*Momordica charantia*), which stood first with 48.7% [5]. OS is renowned for its consumption in the form of a herbal infusion known as Java tea. For ages, OS leaves have been widely used in traditional medicine practices across South East Asian (SEA) region due to its curative effects in diabetes, hypertension, edema, hepatitis, jaundice, renal calculi, gout, and rheumatism. Numerous reports confirming their effectiveness as a cure for nephrolithiasis, hydronephrosis, diabetes, vesical calculi, and arteriosclerosis could be retrieved [6–8].

Few pharmacological reports on the antidiabetic activity of OS in in vivo experimental models are available, whereby hypoglycemic and antihyperglycemic potentials of OS extracts have been reported. Repeated daily oral administration of OS aqueous extract for 14 days at a dose of 500 mg/kg bw elucidated comparable plasma glucose lowering effects with that of 5 mg/kg bw of glibenclamide [9]. Furthermore, it was identified that OS does not potentiate the glucose-induced insulin secretion [10]. However, no studies explored the underlying mechanism of glucose or any other DM biomarker regulation by OS treatment. Although potent inhibitory activity of OS on the pancreatic *α*-amylase and intestinal *α*-glucosidase, two enzymes which cause a sudden postprandial hyperglycemia in type 2 DM by enhancing the hydrolysis of starch and uptake of glucose was suggested [11], until date, the exact protective mechanism of OS in DM is not reported, to the best of our knowledge. Therefore, it is of great significance to investigate the underlying mechanisms of antidiabetic activity to derive deeper insight into their therapeutic effects and thus to ensure its safe and effective usage.

Metabolomics identifies possible metabolic pathways by unraveling the complex inter-relationships of cellular metabolites by their identification and quantification using sophisticated analytical and statistical techniques. Metabolomics has already demonstrated the suitability in the evaluation of the pharmacological effects and mechanism of action of various herbs [12, 13]. As metabolomics involves the quantification of small endogenous molecules such as amino acids, sugars, lipids and organic acids, which are either end products or intermediates in various metabolic pathways, it offers an efficient and simple tool to get a comprehensive snapshot of the internal cellular environment [14, 15]. Furthermore, the compatibility of peripheral fluids such as urine and serum in a metabolomic analysis has promoted its application in pharmacological-toxicological studies by the holistic and global determination of metabolites, or patterns of biomarkers that altered as the result of a stimulus [16]. Although many kinds of advanced instrumental approaches could be utilized in metabolomics studies, Nuclear Magnetic Resonance (NMR) spectroscopy is one of the most preferred methods as it offers simple sample preparation, handling and data analysis techniques [17]. Recently, reports on the use of metabolomics in the study of diabetes with an objective of identifying new metabolic biomarkers and therefore to further the understanding on the underlying biochemical mechanisms and metabolic pathways are in plenty [18–20].

Accumulating all necessary points in an attempt to solve the stated problem, this study was designed to investigate the effect of various standardized OS extracts in the endogenous metabolites of the diabetic rat after 14 days of oral administration, through ^1^H NMR metabolomic analysis of urine samples. Herein, Streptozotocin (STZ) induced rat model was used. The modulatory effects of OS on potential diabetic biomarkers in the rats were investigated and a pathway analysis identified the most relevant therapeutic targets, and thus, the metabolic pathways involved in the treatment, thereby unraveling the mechanism. To the best of our knowledge, this study is the first report on the protective effect of OS on pathological changes of STZ-induced diabetes mellitus based on metabolomic approach, which may serve to fill the knowledge gap about the possible mechanisms and metabolic targets of OS on DM.

## Methods

### Chemicals and reagents

3-Trimethylsilylpropionic acid (TSP) and Streptozotocin (STZ) were obtained from Sigma-Aldrich (St. Louis, USA). Deuterium oxide (D_2_O, 99.9%), deuterated methanol (CD_3_OD, 99.9%), potassium dihydrogren phosphate, deuterated sodium hydroxide and sodium azide were purchased from Merck (Darmstadt, Germany). The normal rat chow feed was purchased from Specialty feeds (Glen Forrest, Australia).

### Preparation of the extract

OS plants of eight to 10 weeks old were collected from Kampung Repuh, Batu Kurau (GPS coordinates: 4.52° N, 100.48° E), Perak, Malaysia in January 2012. After authenticated by a botanist, a voucher specimen (SK1997/12) was deposited at the herbarium of Institute of Bioscience, Universiti Putra Malaysia, Malaysia. The separated leaves were cleaned and then dried using industrial scale continuous drying microwave equipment with an output frequency of 2.45 ± 0.05 GHz for a period of 40 s. The selection of the microwave frequency was based on a trial and error basis in ensuring an efficient drying process that caused little stress to the composition of the leaves as assessed by their color and texture. The dried leaf material was then ground in a blender to powder; size uniformity was ensured by sieving through a stainless steel mesh of 200 mm diameter and stored in airtight containers at 3 ± 2 °C for further processing. The powdered leaf material was then extracted by ultrasonic assisted extraction method. Briefly, weighed quantity of the leaf material was extracted in a measured volume of solvent (ratio of 1 g: 20 mL) by subjecting to sonication for 30 min, while maintaining the sonication bath at a temperature window of 30–40 °C. The extract was filtered before repeating twice with fresh solvent and sonication step, for each time. The filtrate was combined and the solvent was removed using rotary evaporator at 40 °C. The resulted crude extracts of OS (OSE) were lyophilized (extraction yield; water: 14% *w*/w; methanol: 9% *w*/w; ethanol 13%, *w*/w and 50% ethanol water: 8% *w*/w) and kept frozen until use.

### Phytochemical analysis of the OS extracts

The HPLC quantification of two major marker compounds of OS, namely rosmarinic acid and sinensetin, in OS aqueous extract was carried out, in accordance with the methods described in our published report on OS [21].

### Animal experimental design and dose preparation

All the animal experiments were conducted in Animal Biosafety Level – 2 (ABSL - 2) housing complex located at Laboratory of Animal Resource, Universiti Kebangsaan Malaysia (Bangi, Malaysia). A total of 60 male Sprague Dawley (SD) rats, 11 weeks old (225 ± 50 g) were used. The animals were maintained in an air conditioned room at 24 ± 2 °C and acclimatized for 7 days before the experiment. Three rats were housed per polycarbonate cages. The light cycle was maintained at 12 h of light and 12 h of darkness and the rats were allowed free access to food and water.

Rats were randomly divided into 12 groups, with five rats in each group. Six groups were normal rats injected with 0.9% saline for control (N), normal rats treated with OSE water (N-A), normal rats treated with OSE methanol (N-M), normal rats treated with OSE ethanol (N-E), normal rats treated with OSE 50% ethanol (N-50E) and normal rats treated with glibenclamide (N-G). The other six groups were diabetic rats divided into diabetic-control (D), diabetic rats treated with OSE aqueous (D-A), diabetic rats treated with OSE methanol (D-M), diabetic rats treated with OSE ethanol (D-E), diabetic rats treated with OSE 50% ethanol (D-50E) and diabetic rats treated with glibenclamide (DG). All animal handling and experimental protocols were performed in strict accordance with the ethics guidelines approved by Universiti Putra Malaysia Animal Ethics Committee (Approval number: UPM/FPSK/PADS/BR-UUH/00485).

The stock solutions of OSE were prepared separately using 1% CMC as the vehicle. The OSE dose used in the study was 500 mg/kg of the rat body weight (bw) prepared from the respective stock solution and administrated by oral force feed. For glibenclamide, 10 mg/kg bw dose was used and dissolved in DMSO (25 mg/ml). All the doses of extracts and glibenclamide were preserved at 4 °C and used within 3 days.

### Induction of diabetes

The fasted rats were injected intraperitoneally with 60 mg/kg freshly dissolved STZ in 0.9% saline. One week after the STZ administration, the rats with fasting blood glucose concentrations of over 300 mg/dl were considered to be diabetic and used in further experiments. The diabetic rats were then treated for 14 days with 500 mg/kg bw of each extract. The experiment is a modified method described by Sriplang et al., (2007), wherein 14 days has proven to have anti-hyperglycemic effect on rat plasma, without any obvious adverse effects [9]. The positive control, glibenclamide was given in similar manner to that of treatment. For urine collection, rats from all groups were kept in metabolic cages for 14 h of fasting. Each urine sample was collected into 0.1 ml of 1% sodium azide solution and then centrifuged for 10 min at 4 °C, from which the collected supernatant was stored at −80 °C until analysis.

### ^1^H NMR spectroscopic analysis of urine

Urine samples were thawed, centrifuged at 5000 rpm for 10 min and then, 400 μl supernatant obtained from each sample was mixed with 200 μl of phosphate buffer solution (0.1232 g of KH_2_PO_4_), containing 10 mg trimethylsilylpropionic acid sodium salt (TSP) prepared in 10 ml D_2_O with 1.0 M NaOD solution (used to adjust the pH to 7.4), in 5 mm standard NMR tube (Norell, Sigma-Aldrich, Canada). The NMR spectra were recorded using 500 MHz NMR spectrometer (Varian Inova 500, Illinois, USA) at 25 °C with the parameter of pulse width (PW) 21.0 μs (90°) and relaxation delay (RD) 2.0 s. Deuterium oxide was used as the internal lock and TSP was used as the calibration standard and referenced the chemical shift at δ 0.0 ppm.

The 2D NMR spectra such as J resolved (JRES), COSY and HMBC were acquired using Bruker Ascend 700 MHz instrument at room temperature (25 °C). The JRES and COSY spectra were acquired using 4 scans, 1 K data points at 128 increments and a spectral width of 16 ppm in dimensions and a relaxation delay of 2 s. The heteronuclear multiple bond coherence (HMBC) spectra were obtained using 8 scans, 1 K data points, 256 t1 increments at a spectral width of 13 ppm and 220 ppm in the proton and carbon dimensions respectively. The relaxation delay was 1.5 s.

### Statistical analysis of ^1^H NMR spectra

All of the NMR spectra were manually phased, baseline corrected and calibrated to TSP at 0.00 ppm. The chemical shift (δ) region 0 to 10 was reduced to integrated bins of 0.04 ppm width to use in Chenomx NMR software package (Chenomx NMR Suite 5.1 Professional, Edmonton, Canada) for multivariate pattern recognition analysis. The region of the spectra associated with residual water and urea (4.66–5.05 and 5.54–6.0 ppm) were removed. The remaining spectral segments for each NMR spectrum were normalized to the total sum of the spectral intensity to partially compensate for differences in concentrations of the samples. NMR data was then imported to SIMCA-P 13.0 software package (Umetrics, Umea°, Sweden) for analysis and visualization by multivariate statistical methods. Data was mean-centered and Pareto scaled prior to analysis by Principal Component Analysis (PCA) and Orthogonal Partial Least Squares-Discriminant Analysis (OPLS-DA). Data were visualized with the scores plot of the two principal components (PC1 and PC2) in which each point represented an individual spectrum of a sample. The metabolites associated with the group separation were indicated by the corresponding loading plots, in which each point stood for a single NMR spectral bin. The validation and significance of the model were done by using permutation test, CV-ANOVA and R2Y/Q2Y values as and when applicable [22].

The pathway analysis and heat map was generated using Metaboanalyst 3.0, (http://www.metaboanalyst.ca), which is a freely available web-based platform for comprehensive analysis of metabolomic data. The univariate analysis of the integration areas of the metabolites was performed. Kolmogorov-Smirnov test was used to check the normality of the distribution. One-way analysis of variance (ANOVA) was done using GraphPad Prism V 7.0 (GraphPad Software Inc., San Diego, USA), Tukey’s test was chosen as the post hoc analysis method. *P* ≤ 0.05 was considered to be statistically significant and the values were expressed as mean ± SEM.

## Results and discussion

### Phytochemical analysis of the OS extracts

The levels of sinensetin and rosmarinic acid in OS aqueous extract, as determined by HPLC quantification method, were 0.19% and 17.86%, respectively. The LC-MS/MS profile of the OS extracts were already reported by our group [23], which identified the presence of rosmarinic acid, protocatechuic acid, caffeic acid, tetramethoxy chalcone derivatives, ferulic acid, syringic acid, kaempferol methyl ether, succinic acid, protocatechuic acid hexoside, and luteolin.

### ^1^H NMR metabolomic analysis

**Fig. 1:**
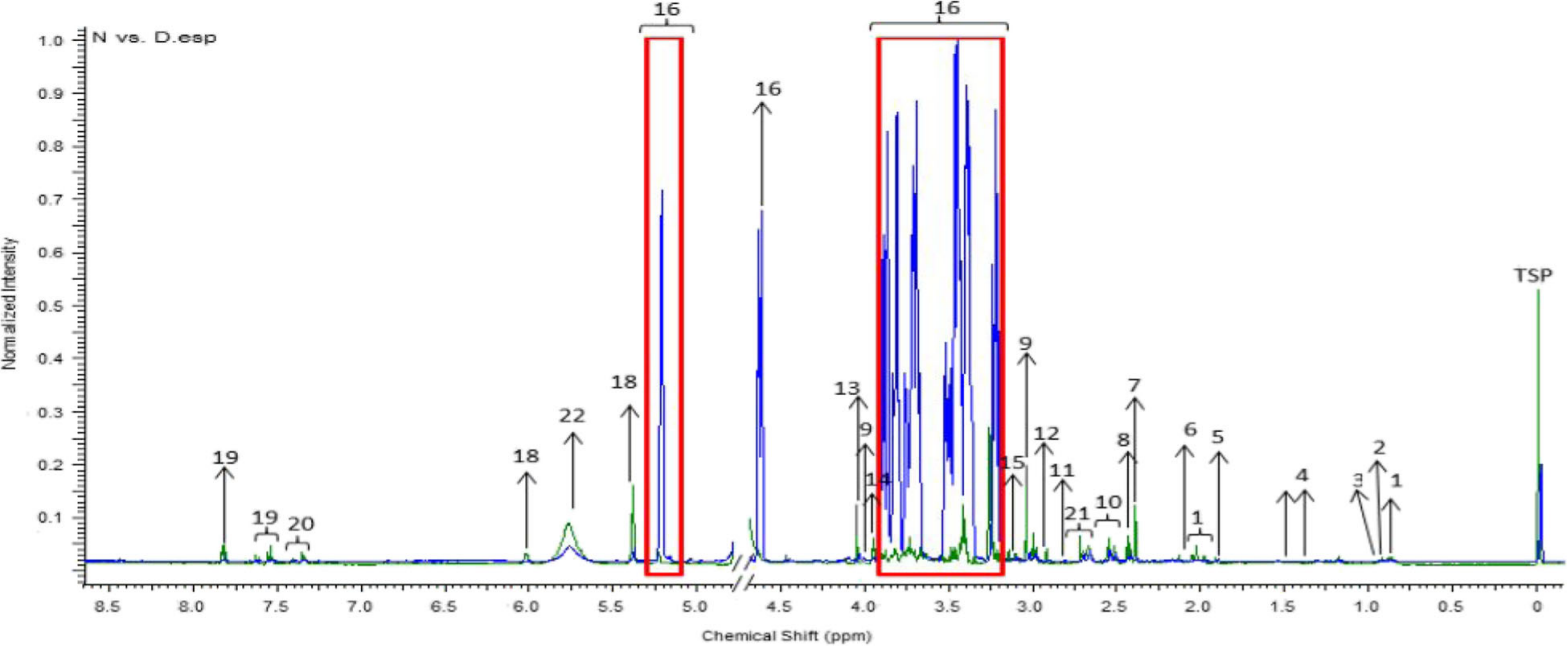
Overlaid ^1^H NMR spectra of normal and diabetic control rats (*Blue*-Diabetes; *Green*-Normal,  Sugar region). (1) Hydroxybutyrate (2) Isoleucine (3) Leucine (4) Lactate (5) Acetate (6) Acetoacetate (7) Succinate (8) Pyruvate (9) Choline (10) Citrate (11) Dimethylamine (12) Creatine (13) Creatinine (14) Betaine (15) Taurine (16) Glucose (17) N-phenylacetylglycine (18) Allantoin (19) Hippurate (20) Phenylalanine (21) Glutamate (22) Urea

The representative ^1^H NMR spectra of rat urine samples obtained from normal, diabetic and OS treatment groups, which respectively represent the characteristic physiological, DM pathological and OSE protective effects are shown in Fig. 1, labelled with identified metabolites. The metabolites assignments were performed based on 2D J RES NMR and COSY analysis, similarity search with Chenomx NMR software database and publicly accessible metabolomic databases such as HMDB (http://www.hmdb.ca), METLIN (http://metlin.scripps.edu) and KEGG (http://www.kegg.jp). A total of 22 metabolites namely hydroxybutyrate, isoleucine, leucine, lactate, acetate, acetoacetate, succinate, pyruvate, choline, citrate, dimethylamine, creatine, creatinine, betaine, taurine, glucose, N-phenylacetylglycine, allantoin, urea, hippurate, and phenylalanine were identified unambiguously. However, urea was excluded in the subsequent analyses as its broad peak of dominating resonance suppresses the nearby peaks. There was no presence of xenometabolomes of the OS extract in the urine spectra from model and treatment groups, perhaps due to their low concentration which made the detection using NMR impossible.

**Fig. 2:**
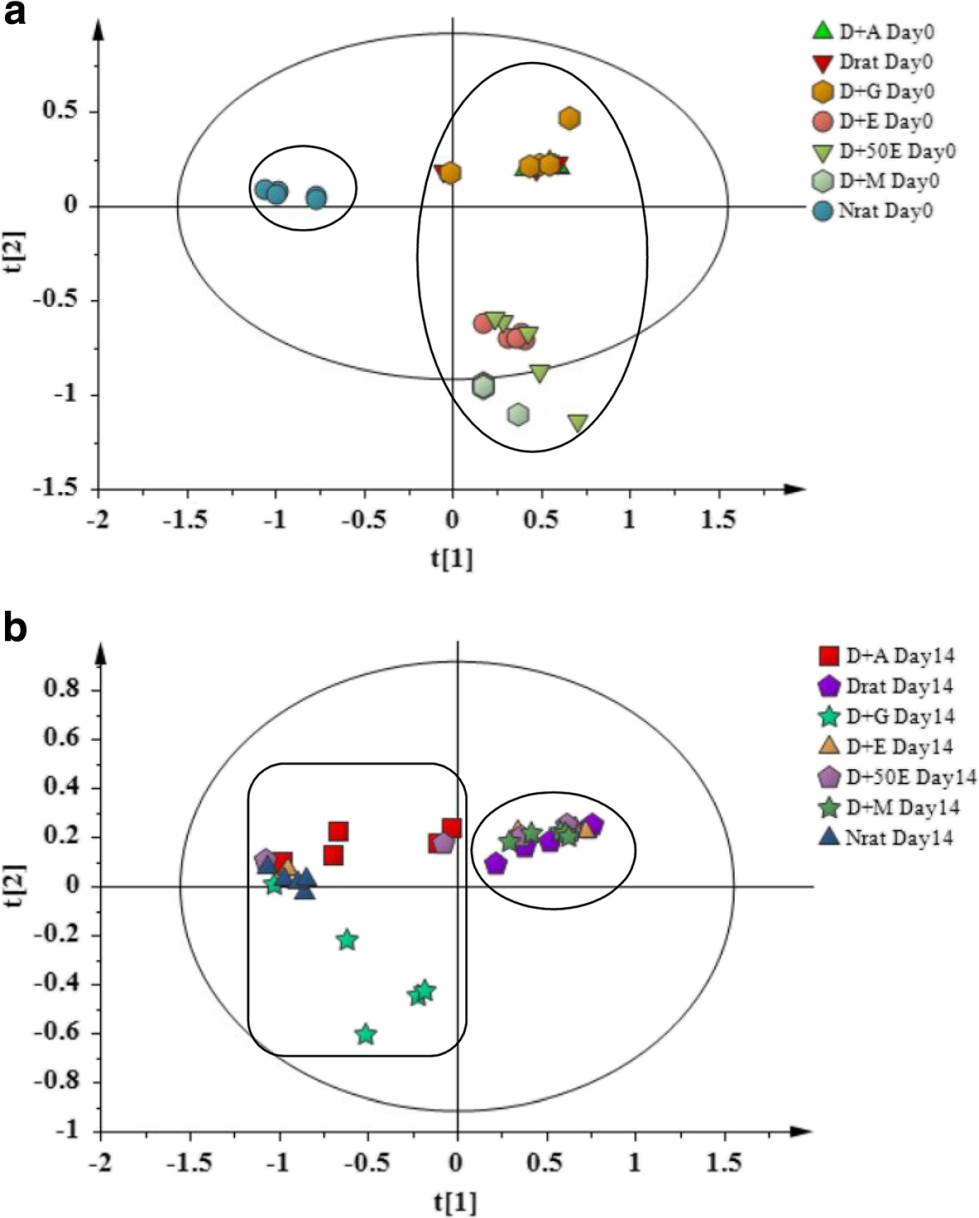
PLS-DA score plot **a** (0 day) and **b** (14 day) based on ^1^H NMR spectra of rat urine

PLS-DA on the ^1^H NMR data of the normal, diabetic and OSE treated groups’ rat urine samples on days 0 and 14 of the experiment provided a global view of the metabolic alterations induced by STZ and OS treatment. The normal group (N) was distinguished clearly from various diabetes groups (D, D-A, D-E, D-50E and D-M) by component 1 on day 0 as demonstrated in score plot (Fig. 2a), which indicated that all of the diabetes groups rats had successfully induced to the diabetic condition. The predictive variation of component 1 corresponded to 48.3% of all variation in the data, with an R2X = 0.99, R2Y = 0.47 and Q2 = 0.11. Whereas, after 14 days OS extracts oral treatment, the diabetic rats treated with aqueous OS was found to be shifted to the negative quadrant of component 1, clustered along with glibenclamide treated rats, and approaching towards the normal rats as evident from Fig. 2b. This indicates that among the various OS extracts, aqueous extract possesses significant antidiabetic activity comparable to glibenclamide.

**Fig. 3:**
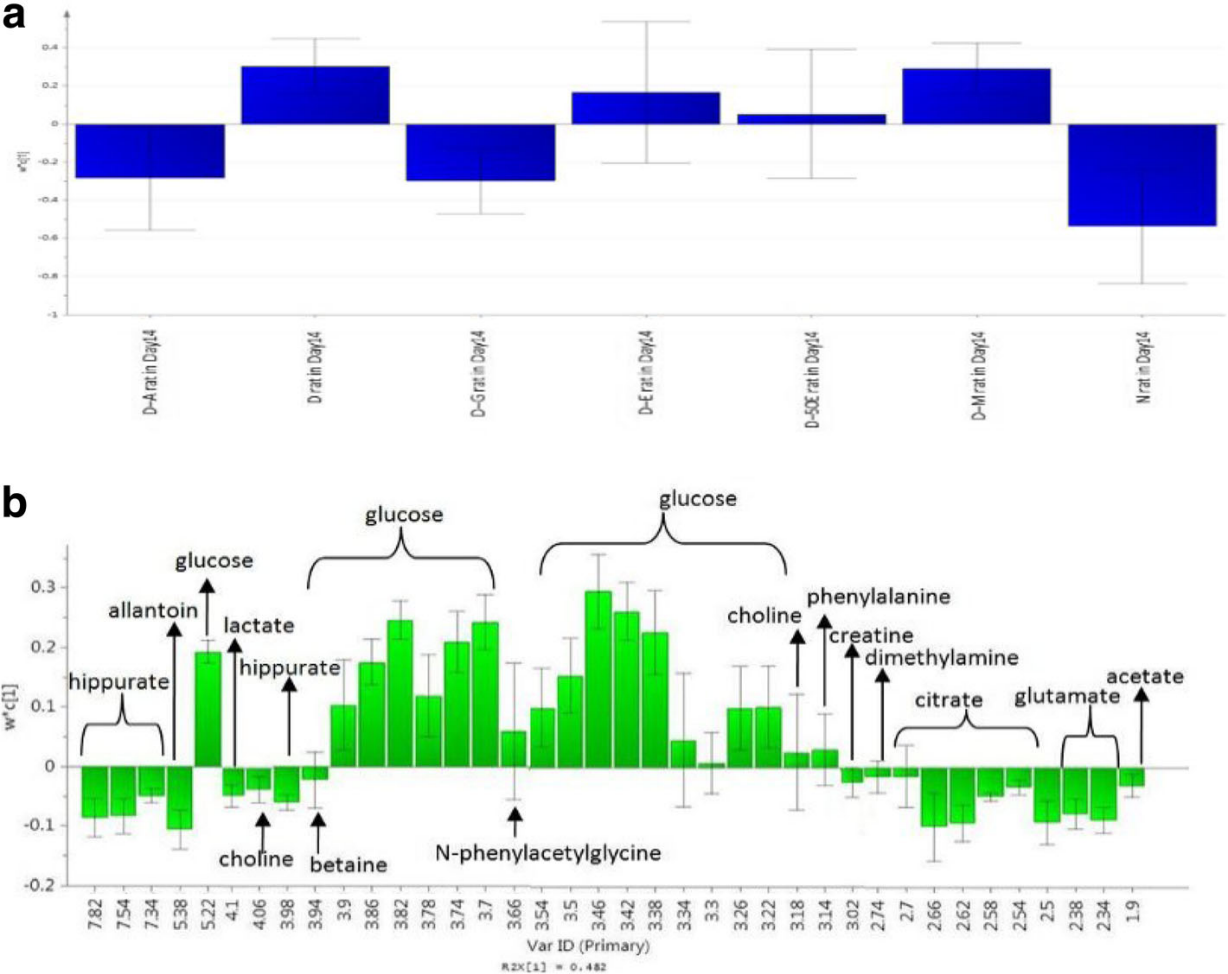
Loading column plot of PLS-DA component 1 in day 14 (**a**) based on class (**b**) based on variables

Figure 3a and b are the PLS-DA loading column plots based on component 1. The variables with negative w*c (1) belong to the classes of N, D-A, and D-G on day 14. This supports that glibenclamide and OSE aqueous (OSAE) have significantly altered the DM marker metabolites so as to approach those of the normal rats. The variables with positive w*c (1) in A belong to all the diabetes groups at day 14, constituted from D, and the OSE ethanol, 50% aqueous ethanol and methanol (D-E, D-50E, and DM) treated groups. Thus, it could be derived that among the four different OS solvent extracts studied, the antidiabetic activity was elicited by only the aqueous extract.

### Opls-DA analysis of the urine samples

**Fig. 4:**
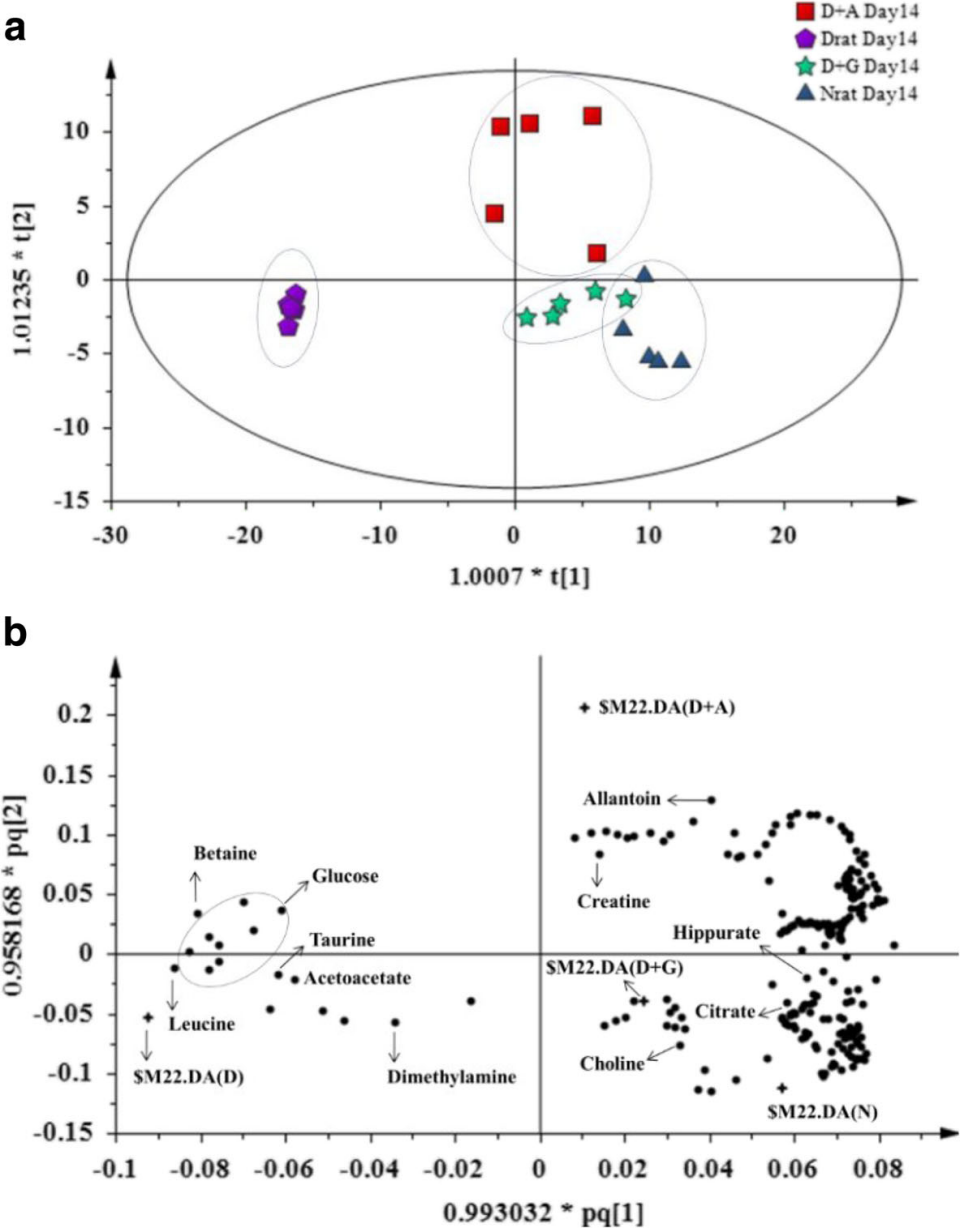
OPLS-DA score plot **a** (14 day) and **b** loading scatter plot based on ^1^H NMR spectra of normal (N), diabetic (D), D-A and D-G rat urine

In order to further unveil the metabolite perturbations caused by OSAE in diabetes, an OPLS-DA was performed on the urine ^1^H NMR spectra between N, D, D-A and D-G groups. The generated OPLS-DA model was subjected to validation using CV-ANOVA, wherein a *P* value of 0.0182 confirmed the validity of the model. PC 1 and 2 together described a total variance of 59.3% with R2Y andQ2 values of 0.875 and 0.647, respectively. As shown in Fig. 4a, the score plot of OPLS-DA revealed a clear discrimination along the t [1] direction between D and N, D-A, D-G groups. The diabetic samples were clustered together on the left side of component 1. The farther clustering of OSAE treatment group from the diabetic group along with its close similarity of the metabolic profile to the normal group suggested the reversal of diabetic injury markers by OS treatment. Glibenclamide treatment group also clustered close to the normal group as the OS treatment group on the right side of component 1. However, the profile of glibenclamide group was closer with the control group compared to OS group. The OPLS-DA findings are consistent with those observed in the earlier PLS-DA model. The corresponding loading scatter plot based on component 1 is shown in Fig. 4b, whereby major metabolites contributing to the separation are revealed. The diabetic biomarkers namely glucose, taurine, acetoacetate, leucine and betaine contributed for the most of the variability responsible for distinct clustering. Interestingly, the glucose level in OSAE treated rats was lowered by 31.4%, while glibenclamide rats by 48.6% after 14 days treatment compared to the diabetic control group. The other metabolites on which OSAE exerted a significant effect were allantoin, creatine, hippurate, choline and citrate.

**Fig. 5:**
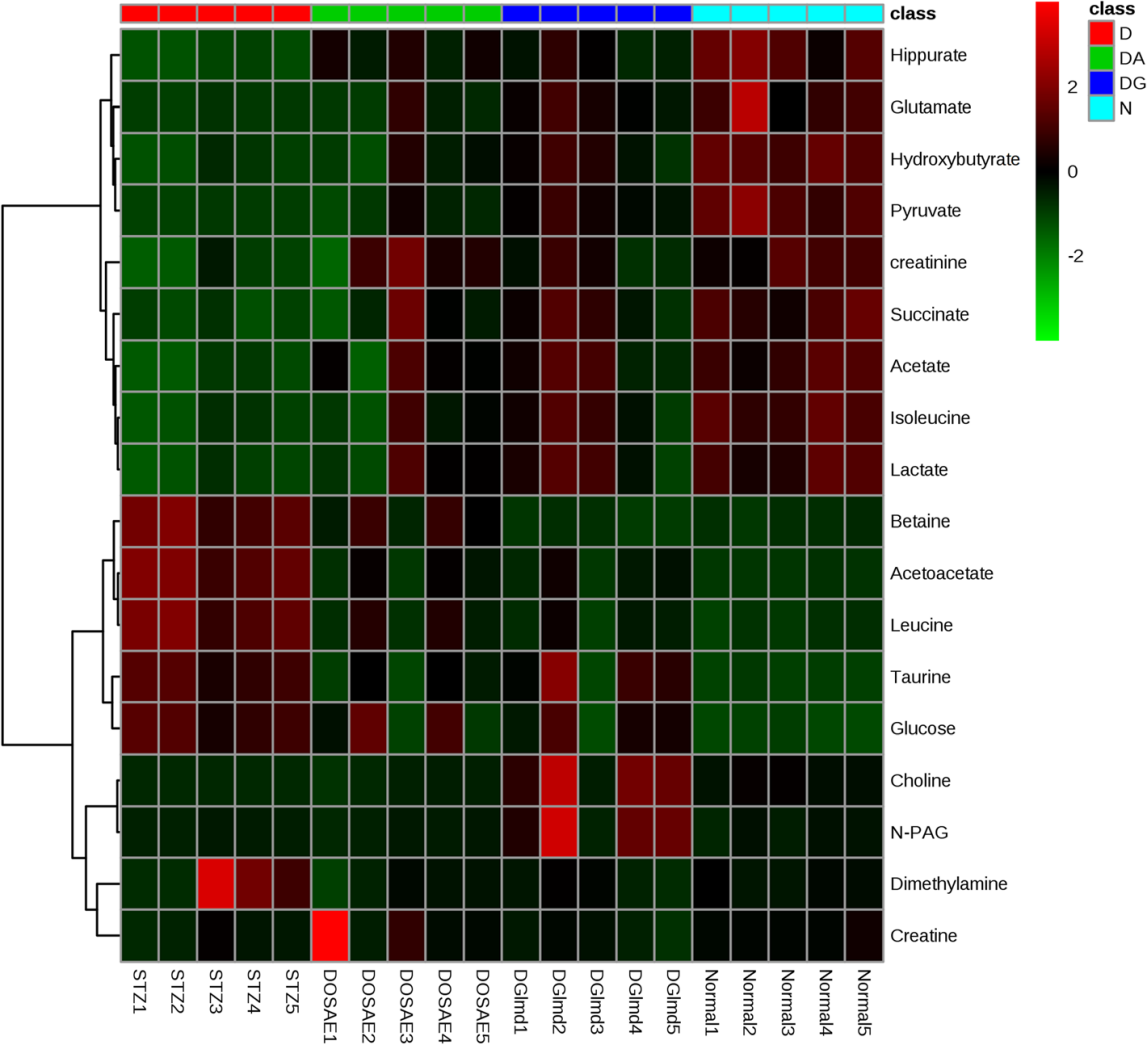
Heat map of the identified biomarkers of Normal, diabetic, D-A and D-G rat urine based on HCA using Ward’s minimum variance method and Euclidean distance. The concentration of each metabolite is colored based on a normalized scale from minimum −3 (*dark green*) to maximum 3 (*dark red*)

**Fig. 6:**
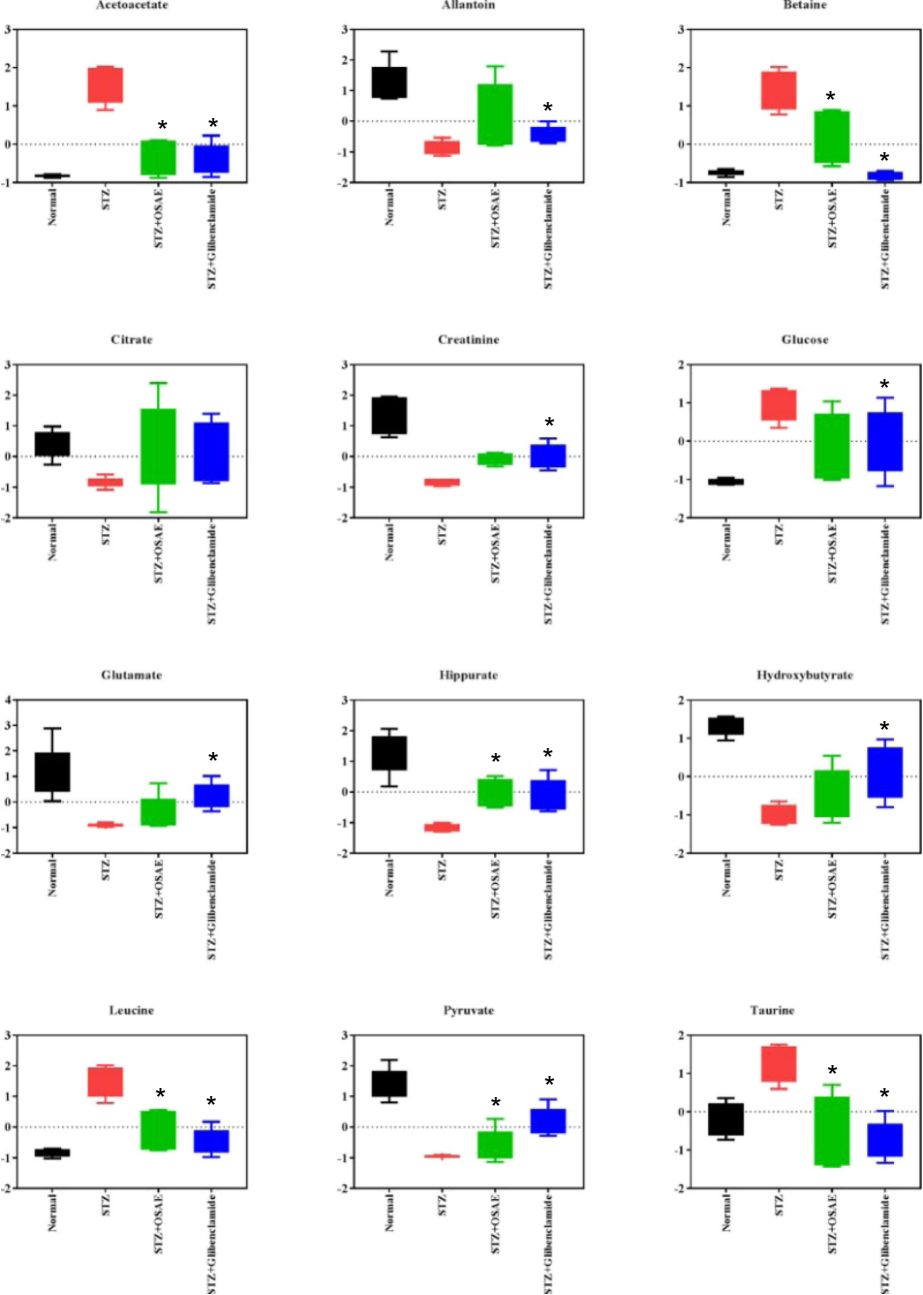
The *box plots* of the relative quantities of the significant biomarkers in the urine samples of normal, diabetic, D-A and D-G rat urine groups

In order to identify the most significantly affected metabolites or the biomarkers of diabetes by the OSAE treatment, metabolite regions were ranked based on the variable importance in projection (VIP) values as given by SIMCA-P 13.0. The VIP value indicates the influence that a particular metabolite exerts on classification, wherein a higher value denotes a higher influence than one with a lower value. The generally accepted notion is that the variables with VIP value >1 exert significant influence on the classification and hence could be treated as biomarkers. From the OPLS-DA model, we identified and characterized a total of 15 endogenous metabolites as the biomarkers. These include acetoacetate, allantoin, betaine, choline, citrate, creatine, creatinine, glucose, glutamate, hippurate, hydroxybutyrate, leucine, N-PAG, pyruvate, and taurine. These findings are in well agreement with the previously reported metabolomics studies on DM [24, 25]. Additional file 1: Figure S1 details the variable region and the respective VIP score of the assigned metabolite. The extent of variation of these identified biomarkers among different experimental groups was understood through hierarchical clustering analysis (HCA). The characteristic binned regions of the 15 biomarkers of importance were normalized and Pareto scaled before subjected to HCA with Euclidean distance measures and Ward’s clustering algorithm. The result of the analysis is visualized as a heat map in Fig. 4, wherein each rectangle represents an averaged binned ^1^H NMR spectral region characteristic of the metabolite of importance which was colored based on a normalized scale from minimum −3 (dark green) to maximum 3 (dark red). The diabetic group recorded significantly increased levels of glucose, taurine, betaine, leucine, and acetoacetate. The OS, glibenclamide treated and normal groups recorded increased levels of metabolites, such as hippurate, allantoin, creatinine, glutamate, hydroxybutyrate, pyruvate, and citrate. Furthermore, an estimation of the changes in these biomarker levels between normal, diabetic, OS and glibenclamide treatment groups were derived through relative quantification, after normalizing to the internal reference (TSP). The box plots of significant individual metabolites are presented in Figs. 5 and 6.

### Metabolic pathways targeted by OS intervention in DM

**Fig. 7:**
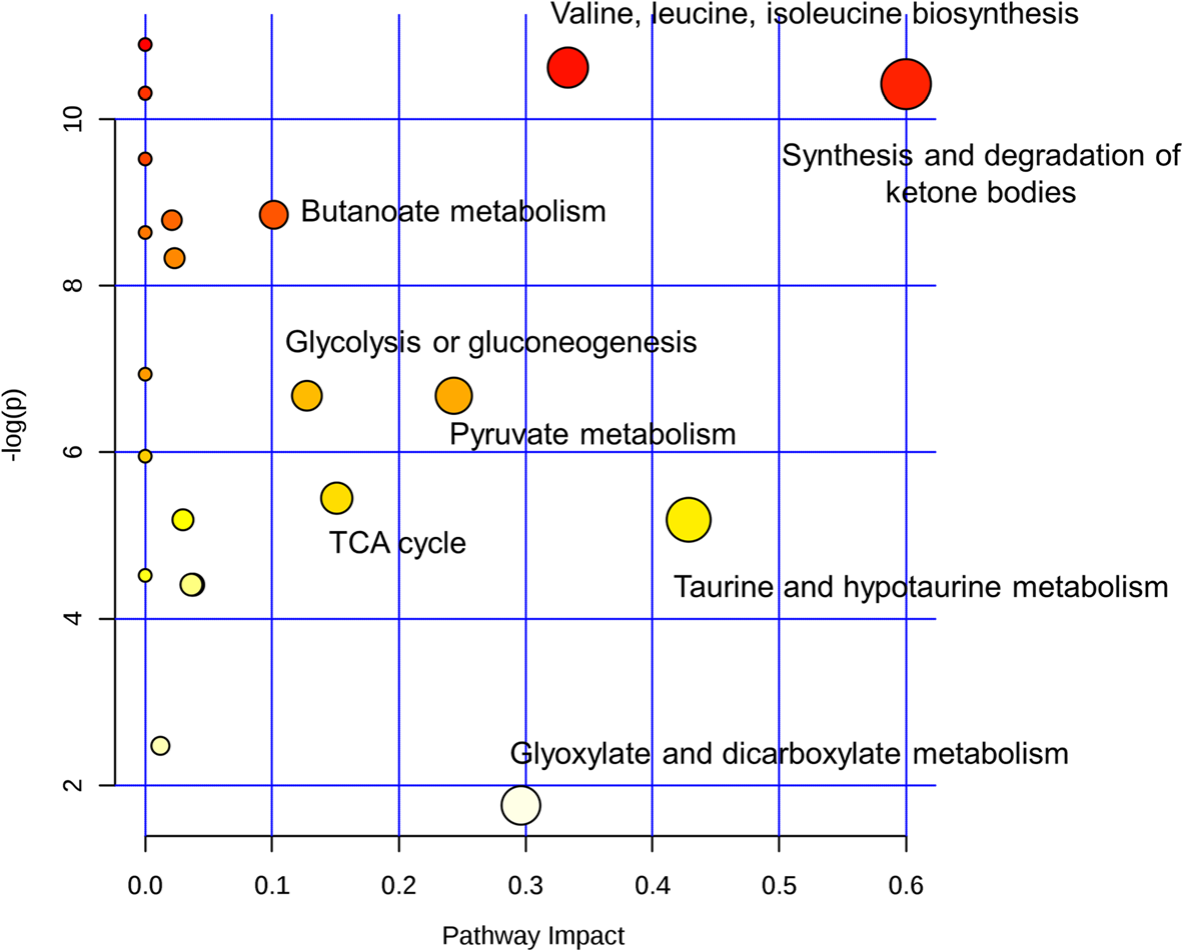
Summary of pathway analysis with MetPA

**Fig. 8:**
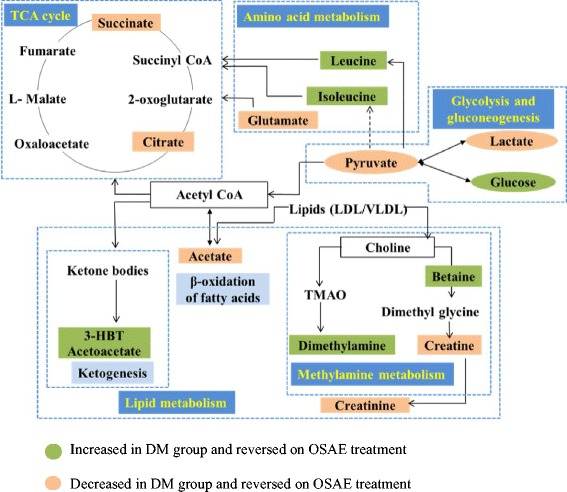
Schematic representation depicting the interrelationships of the disturbed metabolic pathways identified by ^1^H NMR urine analysis

**Fig. 9:**
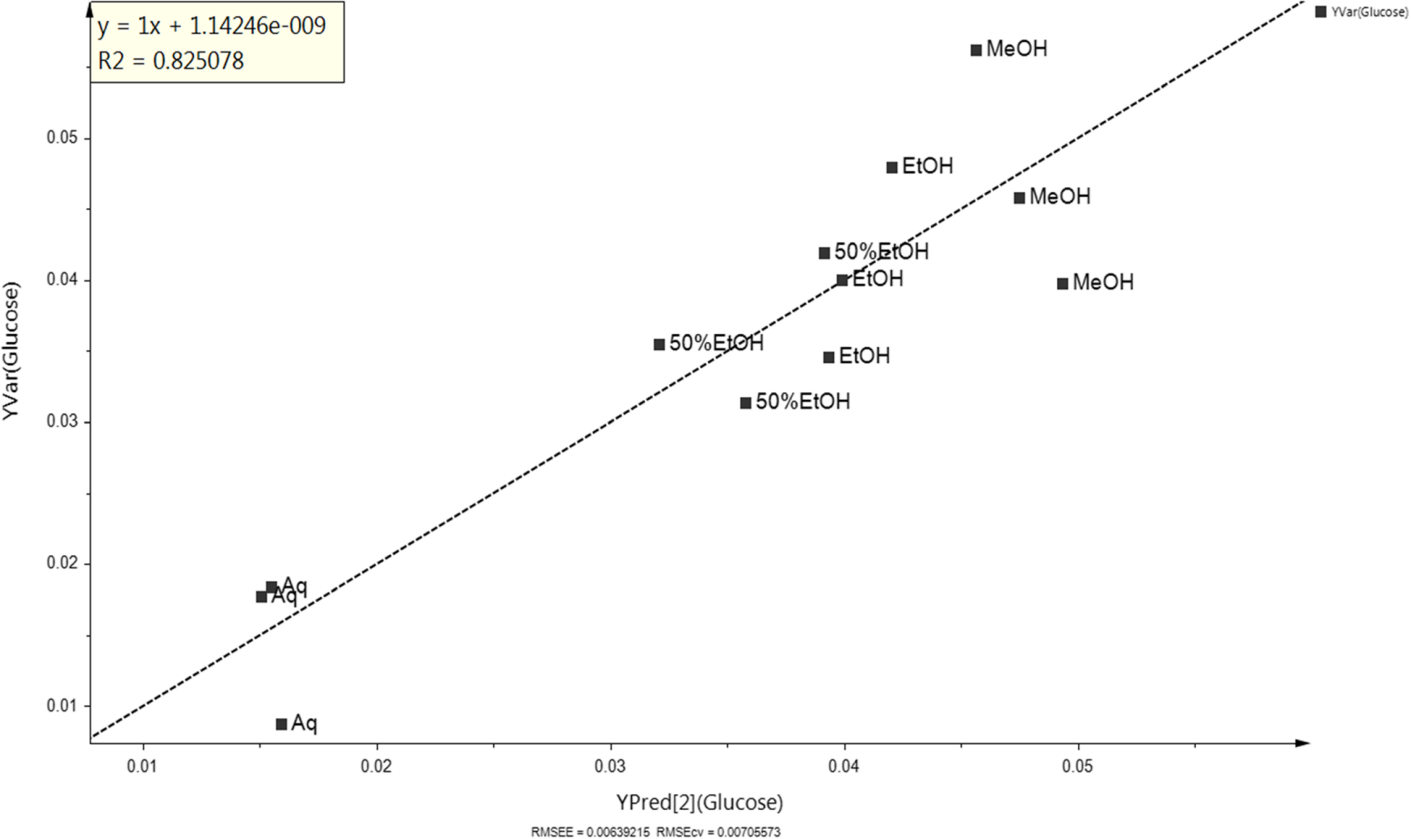
Regression model on bioactivity of extracts towards chemical variable in diabetic and normal rat model treated with OSE in urine

The identified metabolic perturbations by means of ^1^H NMR suggested various metabolic pathways’ alterations between the diabetic and OS or glibenclamide treated rats. Based on the identified biomarkers from the NMR spectral profiles of the urine samples, involvement of several metabolic pathways, namely carbohydrate/glucose metabolism (glucose, pyruvate, lactate), tricaboxylic acid (TCA) cycle (succinate, citrate, hydroxybutyrate), lipid/glycerophospholipid metabolism (acetoacetate, acetate), choline (choline, dimethylglycine), amino acid metabolism (taurine, alanine, hippurate, creatine), methylamine metabolism (dimethylamine) and nucleotide metabolism/purine metabolism (allantoin) could be identified. In order to systematically isolate the most significant pathways involved in the protective mechanism of OSAE in diabetes, the metabolic pathways analysis (MetPA) using MetaboAnalyst (www.metaboanalyst.ca/MetaboAnalyst) was performed. The pathway impact factor, which is a measure of the metabolites’ importance in the network, is used as an index to determine the most relevant pathways [26]. The analysis of the ^1^H NMR data resulted in the identification of 20 metabolic pathways, among which the synthesis and degradation of ketone bodies was the most significant with the highest pathway impact value of 0.6 (Table 1). Based on the set criteria of pathway impact value greater than 0.1, seven other disturbed metabolic pathways were also identified to be highly significant, namely, valine, leucine and isoleucine biosynthesis, butanoate metabolism, taurine and hypotaurine metabolism, glyoxylate and dicarboxylate metabolism, pyruvate metabolism, citrate cycle (TCA cycle), and glycolysis or gluconeogenesis (Figs. 7, 8 and 9). Hence, these pathways represent their potential as the targeted pathways of OSAE treatment in diabetic condition.

**Table 1:** Results of ingenuity pathway analysis with MetaboAnalyst (MetPA)

No.	Pathway name	Hits	Raw P	−log(P)	Impact	^a^FDR
1	Synthesis and degradation of ketone bodies	2	0.000029838	10.42	0.6	0.00017
2	Taurine and hypotaurine metabolism	1	0.005564	5.1914	0.42857	0.00730
3	Valine, leucine and isoleucine biosynthesis	2	0.00002452	10.616	0.33333	0.00017
4	Glyoxylate and dicarboxylate metabolism	1	0.17207	1.7598	0.2963	0.17207
5	Pyruvate metabolism	3	0.001258	6.6783	0.24337	0.00220
6	Citrate cycle (TCA cycle)	3	0.0042902	5.45	0.1510	0.00643
7	Glycolysis or Gluconeogenesis	3	0.001258	6.6783	0.1253	0.00201
8	Butanoate metabolism	4	0.00014336	8.8501	0.10145	0.00045

^a^ *FDR* false discovery rate

### Tricarboxylic acid (TCA) cycle

The reduced levels of tricarboxylic acid (TCA) cycle intermediates, namely citrate and succinate in the diabetic group were found to be restored to nearly normal levels in the OSAE treated rat urine samples. The TCA cycle comprises of various anabolic and catabolic biochemical pathways which modulate the electron transport chains in mitochondria, ultimately resulting in the production of energy by generating ATP [27]. Major metabolic processes of the body such as gluconeogenesis, lipogenesis and amino acid biosynthesis are linked to the TCA cycle activity. The acetyl-CoA, produced from pyruvate (resulted from glycolysis) in a process mediated by pyruvate dehydrogenase, links the utilization of glycogen, glucose, and lactate with the TCA cycle to suffice the energy needs of cells. Thus, increased levels of pyruvate and lactate coupled with that of citrate and succinate confirm that OSAE treatment modulates TCA cycle in order to regulate the blood glucose level, possibly by means of increased glycolysis and decreased gluconeogenesis.

### Glycolysis/Gluconeogenesis

Gluconeogenesis and glycolysis are two metabolic mechanisms responsible for ensuring glucose homeostasis. Glucose metabolizes to produce ATP and NADH, and thus is the metabolite responsible for energy production. Pyruvate is an end product of glucose metabolism through glycolysis, which converts to acetyl-CoA in a process mediated by pyruvate dehydrogenase and enters TCA cycle. Pyruvate production is directly correlated to glycolysis, whereby high glucose level resulted from glycolysis causes reduction in the pyruvate level in diabetes and also stalls further glycolysis [28]. Increasing glucose level inhibits the glycolytic enzymes (phosphofructokinase, pyruvate kinase and hexokinase), thereby hinders the generation of pyruvate. The reduction of pyruvate ends up in reduced formation of Acetyl-CoA and thereby reduces the TCA cycle activity and thus contributes to mitochondrial dysfunction. The results from the present study showed that pyruvate level was increased in OSAE treatment group when compared to the diabetic group. In addition, this could be correlated with the observation that TCA cycle intermediates, namely succinate and citrate, were also increased by OSAE treatment. Hence, OSAE comprehensively restores the mitochondrial function through the mediation of various intermediary metabolites.

### Lipid metabolism

The abnormalities in lipid and fatty acid metabolism cause dyslipidemia, which is one of the main risk factors and complications of diabetes mellitus. The insulin resistance causes to increase the free fatty acids and it eventually shifts the energy metabolism from glucose to lipids (*β* - oxidation of fatty acids) with the aid of acetate. The levels of major ketone bodies such as 3-hydroxybutyrate (3-HBT) and acetoacetate could be correlated to the extent of fatty acid metabolism in the liver, as they constitute the representative metabolites. The increased level of acetoacetate and 3-HBT in diabetic rats indicated activation of ketogenesis pathway and hence the mitochondrial dysfunction in diabetic rats [29]. Compared with diabetic rats, OSAE treated rats had shown a remarkable reversal in the acetoacetate and 3-HBT levels suggestive of the re-establishment of energy metabolism via glucose regulation.

### Amino acid metabolism

The levels of branched-chain amino acids (BCAA) such as leucine and isoleucine were increased in the urine of diabetic rats. In diabetic conditions, other than lactate, these ketogenic amino acids act as gluconeogenic precursors, resulting in the lactate accumulation in the blood [30]. OS treatment effected partial reversal of the elevated BCAA and, therefore, it hinted at the suppression of ketogenesis or gluconeogenesis, which further supports the metabolic alterations observed under lipid metabolism. Furthermore, the increased amount of amino acids in diabetic rats suggested perturbed protein synthesis, which could be the reason for the observed weight loss [31]. OSAE treatment had an obvious regulatory role on amino acid metabolism as observed from the fact that the reduction in body weight of OS treated group was lower than that of diabetes group (Additional file 1: Figure S2).

### Regression model

A validated regression model was built in deciding whether the bioactivity of a new set of OS extract could be hypothesized in relation between variables obtained from the regression analysis, without the necessity of having to do the bioassay [32]. The regression model, as represented by the data, was validated based on the coefficient of determination (R2 = 0.825) value, whereby most of the data lie near to the linear regression line of best fit line [32]. The obtained ^1^H NMR data of the plant extracts were used as the X variables and the relative concentration of glucose was used as the Y variable. All OS extracts exhibited alteration of the glucose level in urine, wherein aqueous (Aq) extract, which is located at the lowest point of Y predicted (Q2), gave the most significant change, followed by 50% ethanolic (50%EtOH), ethanolic (EtOH) and methanolic (MeOH) extract.

## Conclusions

A ^1^H NMR based urine metabolomics tool has been used for the first time to evaluate the protective effects of OS in DM using STZ induced experimental model in rats. Pattern recognition combined with multivariate statistical analysis identified that 14 days oral administration of OSAE at the dose of 500 mg/kg bw caused the reversal of DM. A total of 15 metabolites, which levels changed significantly upon treatment were identified as the biomarkers of OSAE in diabetes. These biomarkers suggested the involvement of several metabolic pathways, whence, a systematic metabolic pathways analysis identified that OSAE exerted the antidiabetic activity through regulating the TCA cycle, glycolysis/gluconeogenesis, lipid and amino acid metabolism. Thus, metabolomics approach aided in exploring the effects of OS in DM biomarkers and provided a better understanding of the mechanistic pathways involved, and proved as a promising tool in the ethnopharmacological validation and mechanism of action studies in traditional medicine research.

## Additional file

Additional file 1: Figure S1. VIP score and variable regions of the assigned metabolites. **Figure S2.** Body weight of rats in day 0, day 7 and day 14 of treatment. Data are expressed as mean ± S.D.; *n* = 5 rats per group. N = normal rat, D = diabetic rat, DG = diabetic rat treated with glibenclamide, D-E = diabetic rat treated with OS ethanol extract, D-A = diabetic rat treated with OS water extract. **p* ≤ 0.05 versus normal control in same day, ***p* ≤ 0.05 versus day 0 and other day in same group. (PDF 243 kb)

## Abbreviations

ANOVA: Analysis of variance
BCAA: Branched chain amino acids
bw: Body weight
CAM: Complementary and alternative medicine
DM: Diabetes mellitus
MetPA: Metabolic pathways analysis
NMR: Nuclear magnetic resonance
OPLS-DA: Orthogonal partial least squares-discriminant analysis
OS: *Orthosiphon stamineus*
OSAE: OS aqueous extract
OSE: Crude extracts of OS
PC: Principal component
PCA: Principal component analysis
SEA: South East Asian
STZ: Streptozotocin
TCA: Tricarboxylic acid cycle
