# Supplementary material for: Urinary metabolomics study on the protective role of Orthosiphon stamineus in Streptozotocin induced diabetes mellitus in rats via 1H NMR spectroscopy
Source: BMC Complement Altern Med. 2017 May 25;17:278. doi: 10.1186/s12906-017-1777-1 (PMC5445454; doi:10.1186/s12906-017-1777-1)
Supplement: Additional file 1: Figure S1. — VIP score and variable regions of the assigned metabolites. Figure S2. Body weight of rats in day 0, day 7 and day 14 of treatment. Data are expressed as mean ± S.D.; n = 5 rats per group. N = normal rat, D = diabetic rat, DG = diabetic rat treated with glibenclamide, D-E = diabetic rat treated with OS ethanol extract, D-A = diabetic rat treated with OS water extract. *p ≤ 0.05 versus normal control in same day, **p ≤ 0.05 versus day 0 and other day in same group. (PDF 243 kb) [file 12906_2017_1777_MOESM1_ESM.pdf]

Additional file

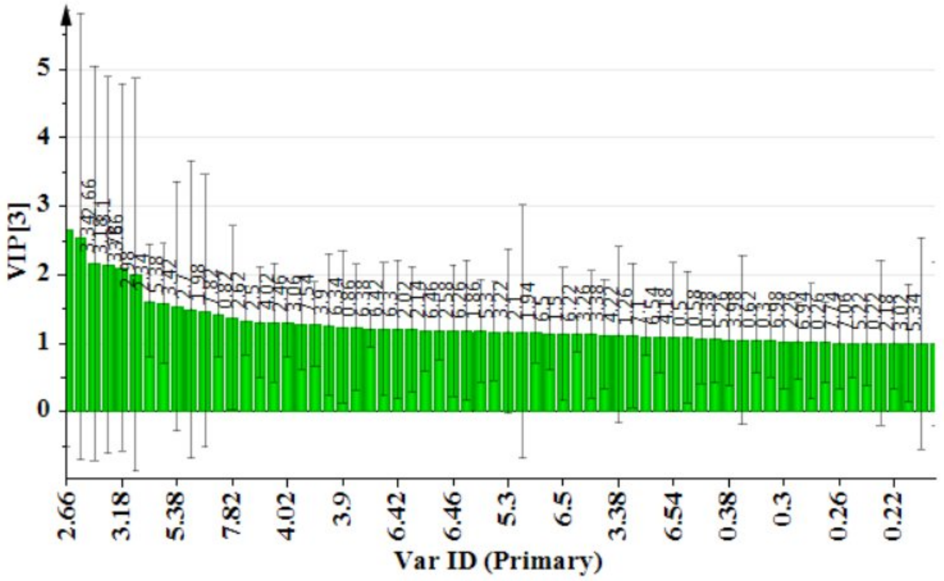

Figure S1. VIP score and variable regions of the assigned metabolites

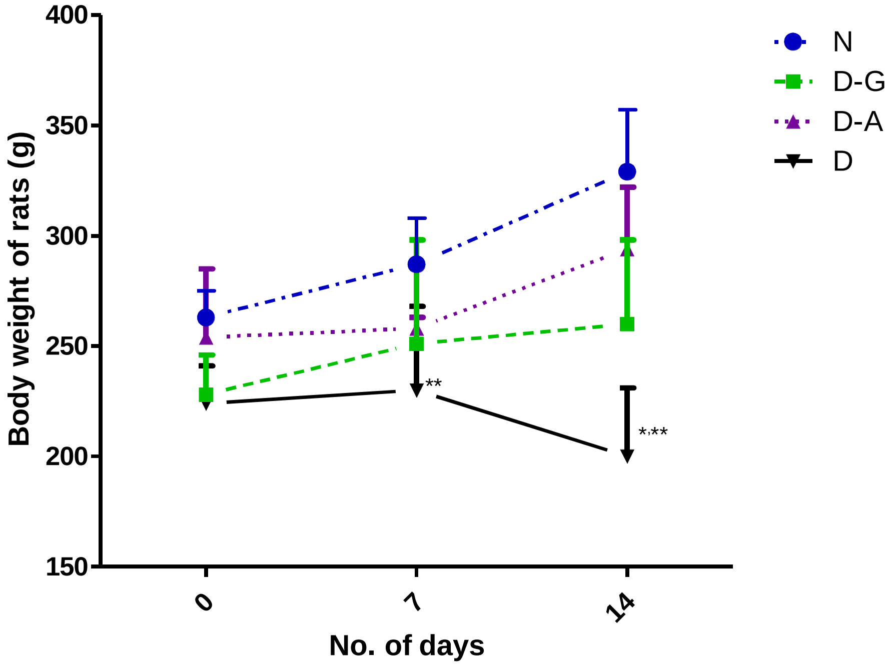

Figure S2. Body weight of rats in day 0, day 7 and day 14 of treatment. Data are expressed as mean ± S.D.; *n*=5 rats per group. N= normal rat, D = diabetic rat, D-G= diabetic rat treated with glibenclamide, D-E= diabetic rat treated with OS ethanol extract, D-A= diabetic rat treated with OS water extract. *p≤0.05 versus normal control in same day, **p≤0.05 versus day 0 and other day in same group.
